# Virtual microscopy with Google-Earth: a step in the way for compatibility

**DOI:** 10.1186/1746-1596-8-S1-S11

**Published:** 2013-09-30

**Authors:** Luis Alfaro, Maria Jose Roca, Pablo Catala

**Affiliations:** 1 Department of Pathology. Fundacion Oftalmologica del Mediterraneo. Valencia. Spain; 2 Department of Pathology. Hospital Arnau de Vilanova. Valencia Spain; 3 IT department. Fundacion Oftalmologica del Mediterraneo. Valencia. Spain

## Background

Advances in the field of virtual microscopy are continuously growing. Many companies have introduced equipments with very good image quality levels, and increased speed in the scanning processes. However also a wide variety of image formats, software viewers and servers have appeared, with lack of compatibility in the managements of virtual slides.

Commercial solutions for virtual microscopy tend to be rigid and difficult of customize, probably to protect the developments, but this leads to a detachment in the management of the images and a difficulty in becoming familiar with this technology. Handling virtual slides in a similar way as we do with conventional pictures taken from digital cameras surely would bring to virtual microscopy a much wider number of pathologist.

In this approach we tried to adapt to virtual microscopy simplistic solutions employed in digital photography as software oriented for panoramic images [1]. Panoramic images share with virtual slides their huge size and a similar way to be generated stitching smaller images [2]. Software for panoramic images can be adapted for virtual microscopy.

Google Earth is a well-known software oriented as a geographic information system working in a way similar to virtual microscopy, zooming and panning images, and moving along huge files. It is widely distributed, installed in may computers and can be useful to share virtual slides and employed as a viewer of virtual slides [3].

There are developments for virtual microscopy based in the use of the API (application programming interface) of Google Maps, such as the NYU School of Medicine Virtual Microscope [4]. These options of high complexity require a team of programmers and computer support, not available in all situations. However, it is possible for pathologists to use Google Earth as a viewer of our virtual slides more easily, and without programming knowledge.

## Material and methods

With the aim of testing the value of Google Earth as a software for the handling of virtual slides we selected 20 pathology cases. Glass slides of 10 were scanned with a·3D-Histech Panoramic Midi, and the other 10 slides with an Aperio XT. Original virtual slides were exported into .jpg flat files with Aperio ScanScope software. We generated kml files, the file format to display information in Google Earth, and the compatible pyramidal tiles structure.

The software employed was GDAL, an open source library for geospatial data images, with the utility gdal2tiles, and its graphical interface variant (MapTiler). All the cases were uploaded into two servers, our own server at the hospital, and a external sever hosting web pages. HTML web pages were built linking the cases with the kml files. MIME types were defined in both servers in order to lead kml files be opened with Google Earth.

## Results and discussion

Cases were accessible anywhere from the Internet through its web address and opened directly with Google Earth. All functions to allow diagnostic, consultation, educational... purposes were available. Image quality obtained was equivalent to any other specific viewer for virtual slides. Speed in serving files was related with lines capacity and hosting server performance, and not with software.

Google Earth uses a specific file type (KML / KMZ) to define specific locations. kmz files are compressed versions of the kml with zip compression. When clicking on these files Google Earth opens and shows the location defined in the file with specific spatial coordinates.

KML files have many features, which can be reviewed at the program tutorials [5]. They allow among them, to insert pictures that are embedded on the landscapes of Google Earth. This feature, called "PhotoOverlay" is often used associated with Google Earth position marks, but also supports the use of very large photographs, with many megapixels as used in our virtual slides. The procedure for visualization of these giant photographs is the usual breaking them into small portions, and arrange them in a pyramidal structure. Each image of the pyramid is divided into tiles, so that only the parts wished to see need to be charged at every moment, and with the known functions of zoom and panning.

Kml files have a syntax similar to html files. They can be created manually and adding a link to the virtual slide with the PhotoOverlay tag is not difficult. Anyway there are available kml file generators [6,7].

The organization of the virtual slide in a set of pyramidal tiles although can also be generated manually, needs in practice an automated system to avoid a long and repetitive process. Several tools are designed for this purpose. We selected MapTiler [8] because of its easiness of use and the simultaneous generation of the associated kml file. The main handicap of this software is that it becomes very slow even in the most modern computers when facing very big virtual slides.

A similar alternative for the generation of the pyramidal image is gdal2tiles.py a software that generates a directory with the small tiles from the fragmentation of the virtual slide an also the kml file to be opened with Google Earth [9]. It’s a command line software based on python programming language not as easy to be used.

**Figure 1:**
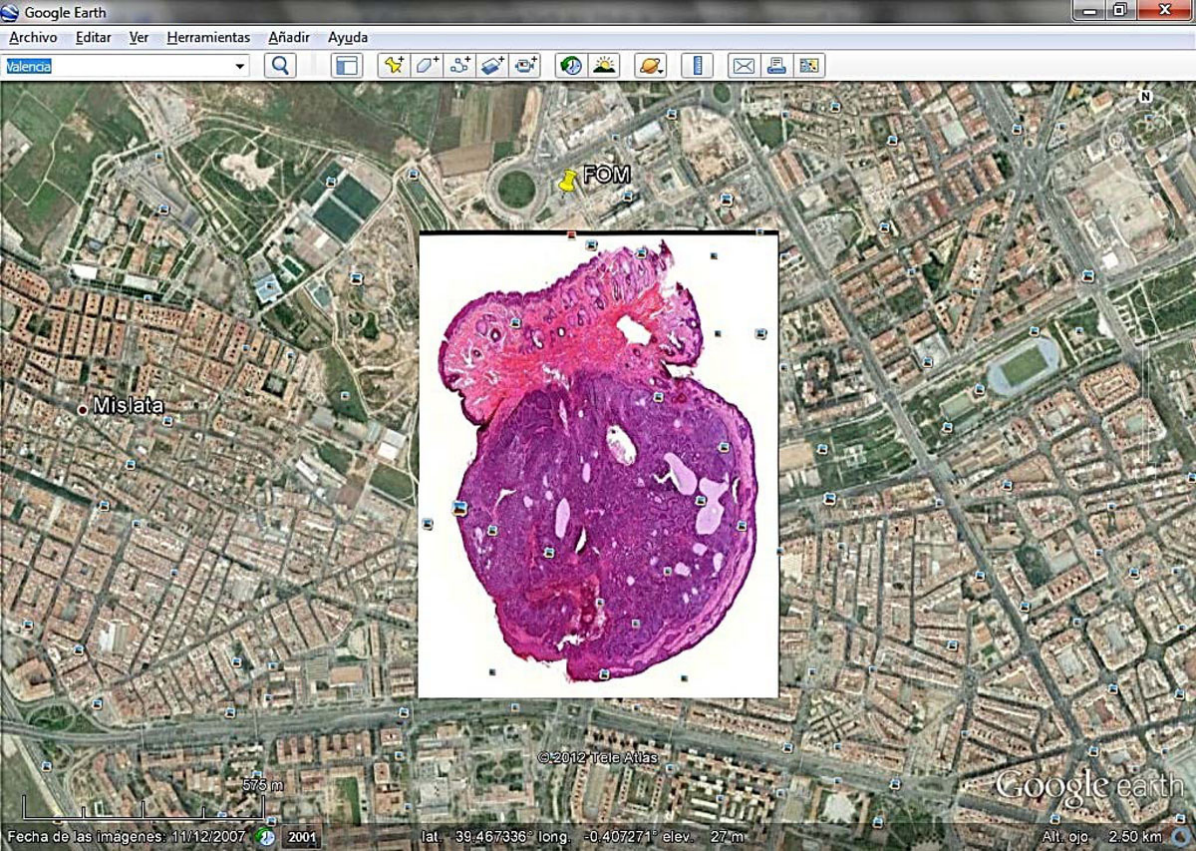
Google Earth showing a virtual slide

An example of a virtual slide prepared to be seen with Google Earth is provided in Figure 1.

Table 1 shows the syntax of the simplified KML file prepared for the example.

**Table 1:** KML file prepared to open a virtual slide with Google Earth

A certain limitation in the use of this alternatives is that original virtual slides need to be exported into flat jpg files which will be the template to generate the pyramidal structure suitable for Google Earth. The well know restriction of jpg files to a maximum size of 65.000 pixels (2^16^) can be insufficient for big virtual slides. JPG2000 format is not available for the analyzed software, and conventional Tiff files have also a size limitation of 4 GB.

## Conclusions

Google Earth is widely distributed and can be a good choice to avoid compatibility limitations in virtual microcopy. Even most viewers for virtual slides are free, sometimes especially for remote consultation, it is not possible to expect a remote pathologist having installed all different browsers. Besides many pathologist in hospitals have not administrations rights to install software at their computers. Viewing slides with Google Earth requires not technical skills and any pathologist can use it easily. Exporting virtual slides and generating the tiles and files to serve them for Google Earth requires a bit more of knowledge in information technologies, however it is possible for a pathologist with some experience in virtual microscopy to do without technical assistance. No programming abilities are needed further the generation of html files to link the slides in the servers, and the full process can be semi-automated. Google Earth is also a very dynamic software with frequent actualization, and many working groups introducing new improvements and can be suitable to be used in virtual microscopy.

## List of abbreviations

GB: Gigabyte; GDAL: Geospatial Data Abstraction Library; HTML: HyperText Markup Language; JPG (JPEG): Joint Photographic Experts Group; KML: Keyhole Markup Language; MIME: Multipurpose Internet Mail Extensions; TIFF: Tagged Image File Format

## Competing interests

The authors declare that they have no competing interests.

## Authors’ contributions

LA Conception, design and initial manuscript writing

MJR manuscript reviewing and important intellectual contribution

PC Technical advice and support
